# A Case Report on a Recurring Rash: Mild Chronic Superficial Perivascular Dermatitis With Epidermal Ulcerations

**DOI:** 10.7759/cureus.78696

**Published:** 2025-02-07

**Authors:** Elizabeth Thai, Maro Valladares, Kim Nguyen, Marai Roque, Mohammed Siddiqui

**Affiliations:** 1 Family Medicine, Chino Valley Medical Center/American University of Antigua, Chino, USA; 2 Family Medicine, Chino Valley Medical Center, Chino, USA

**Keywords:** dermatitis, perivascular, rash, skin, superficial ulcerations

## Abstract

This case report presents a 53-year-old male with a five-month history of ongoing purpuric, blistering, and ulcerative rash on his bilateral lower extremities that extended upward to his torso, back, and upper extremities. Although the patient was placed on numerous antibiotics, the rash persisted and continued to cause the patient distress. During his multiple hospital admissions, repeated wound cultures grew* Enterobacter aerogenes*, *Enterobacter cloacae*, *Escherichia coli*, *Klebsiella oxytoca*, and recurrent methicillin-resistant *Staphylococcus aureus* (MRSA). Skin biopsy showed mild chronic superficial perivascular dermatitis with epidural ulceration and no evidence of leukocytoclastic vasculitis. This case report highlights the difficulties in the management of complicated cases of dermatitis and the importance of quick diagnoses, compliance, and outpatient follow-up.

## Introduction

Superficial perivascular dermatitis is an inflammation of the skin characterized by inflammatory cells surrounding blood vessels. These cells can cause damage via invasion into the vessel, fibrosis, or causing necrosis [1,2]. There are different causes and subtypes within the umbrella term of perivascular dermatitis. Some examples include urticaria which is predominantly lymphocytic in nature with a usually normal-appearing epidermis, macular amyloidosis which consists of eosinophilic globules in the papillary dermis and is also associated with melanophages, erythema annulare centrifugum which resents with sharply demarcated arrangements of perivascular lymphocytes with minimal intervascular extension, Shamberg’s disease which is the most common type of pigmented purpuric dermatoses associated with hemosiderin and extravasated erythrocytes and is associated with venous stasis, and viral exanthem which is morbilliform or maculopapular in nature and is associated with extravasated erythrocytes and minimal epidermal changes [3,4]. Superficial perivascular dermatitis normally does not include epidermal changes or epidermal changes are minimal if present. If there are epidermal changes, there is likely a secondary diagnosis. The patient we will be discussing suffers from both superficial perivascular dermatitis and ulcerations, likely meaning there are underlying causes as to why he has concurrent ulcerations. Chronic superficial perivascular dermatitis with epidermal ulcerations and other related differentials are particularly interesting for presentation based on its unusual clinical presentation. Often the evolving disease or early pathology and its unique etiology can change the symptoms or presentations at different stages causing increased difficulty in diagnosing. This is a broad topic that can be difficult to diagnose because of the wide range of causes and overlapping signs and symptoms, making it hard to narrow down differentials, with this information we can have more implications to broaden our clinical practice.

## Case presentation

A 53-year-old male with a past medical history of hypertension, not on any medications presented to the ED on February 9, 2024, with an oozing wound to his right lower extremity that he had noticed about one month ago. Three weeks prior, he noticed a rash spreading upward on both lower extremities, his torso, back, and upper extremities. He finished a course of oral and topical antibiotics from his primary care provider but noted no improvement in the wound. Associated symptoms included fatigue, loss of appetite, pain, and burning. He also reported one episode of gonorrhea 20 years ago that was treated with no complications. He had not been sexually active for about a year; his last encounter was with a woman. He was recently tested for HIV and reported that it came back negative. To his knowledge, he was up to date with his vaccinations. Previous surgeries included right ankle fracture fixation and right shoulder laceration repair. He has a 26-pack-year smoking history and admitted to using crystal meth.

**Figure 1:**
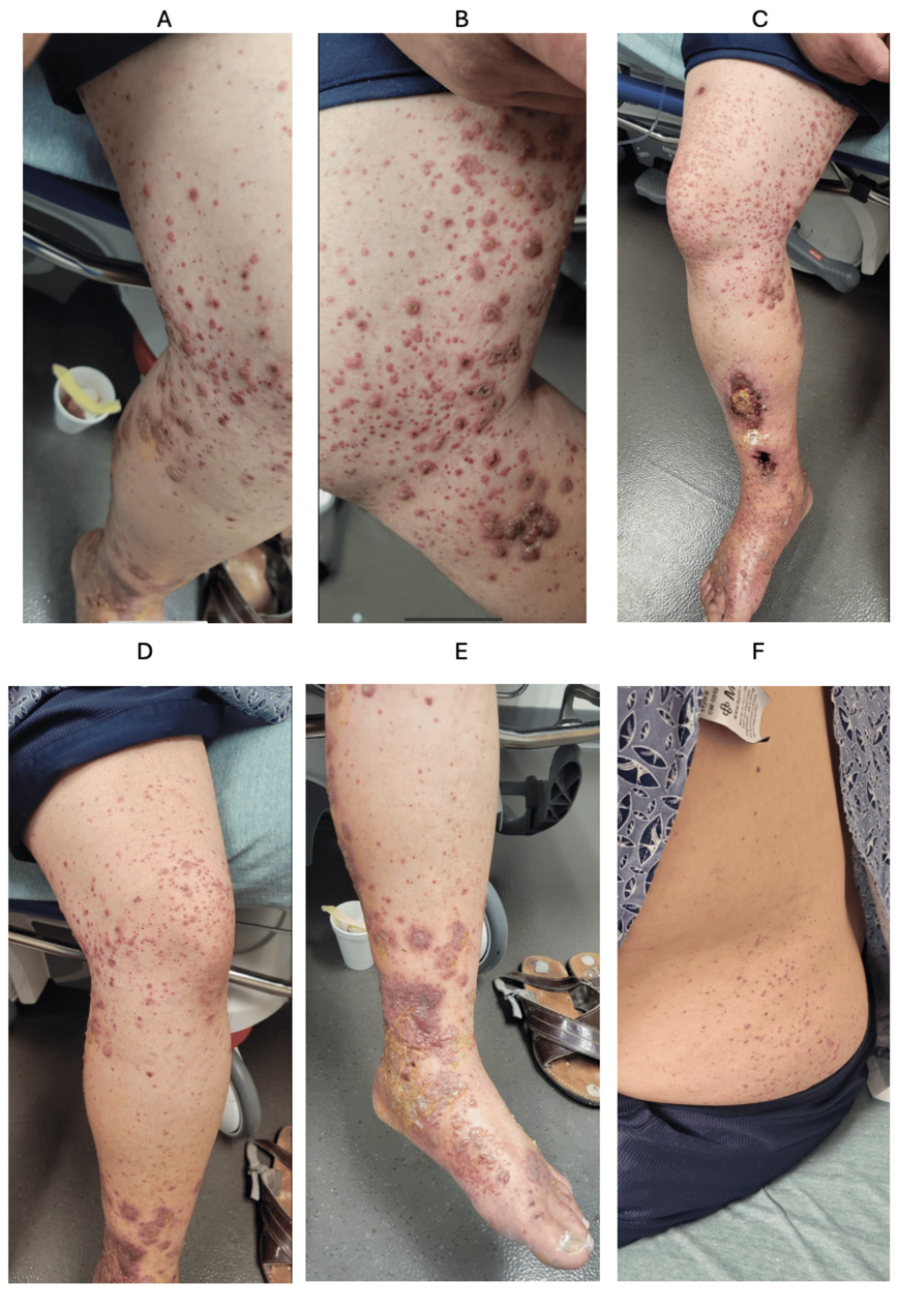
Patient's photos taken during physical exam (A) Purpura on the medial side of the left lower extremity. (B) Purpura, crusting, and blistering on the medial side of the right lower extremity. (C) Purpura to the proximal portion of the right lower extremity. Open ulceration to the distal portion of the right lower extremity. (D) Purpura to left lower extremity. (E) Purpura, crusting, and blistering to the distal portion of the left lower extremity. (F) Purpura to left flank area.

On physical exam, he had a petechial rash and palpable purpura that extended from the feet to the thighs bilaterally and up to his lower back and torso (Figures 1A-1F). There were areas of crusting and ulcerations, as well as blistering with clear discharge noted.

**Figure 2:**
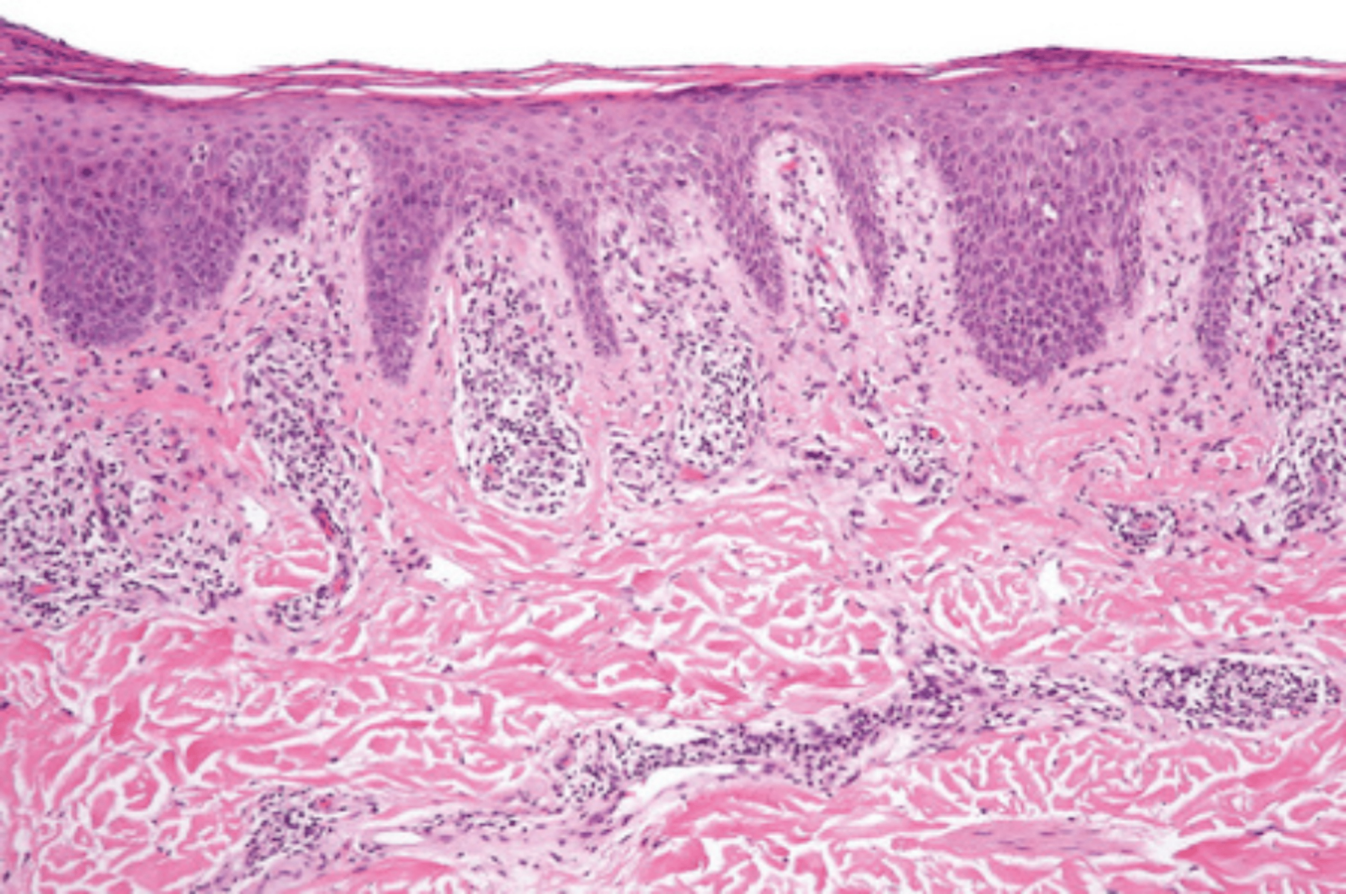
Example of biopsy of superficial perivascular dermatitis at 20x magnification

Labs were done daily during his admission to monitor trends (Tables 1-6). On admission, the patient was found to have mild normocytic anemia, an elevated d-dimer and fibrinogen, hypokalemia, acute kidney injury, and an elevated CRP. The hepatitis panel and HIV test were both negative. During his admission, he did develop mild leukocytosis, which may have been due to steroids given, prednisone 40mg. A venous duplex ultrasound of the bilateral lower extremities ruled out a DVT but could not exclude an isolated calf vein thrombosis. A renal ultrasound showed a left renal cyst, debris within the bladder, a markedly enlarged heterogenous prostate gland, and no evidence of hydronephrosis. Wound and blood cultures were taken and later methicillin-resistant *Staphylococcus aureus* (MRSA) and *Enterobacter aerogenes* in the wound. An infectious disease doctor was consulted for this case and suspected vasculitis possibly with superimposed infection. The patient was started on Vancomycin and Ceftriaxone. This doctor suggested taking biopsies of the lesions and to consider a dermatology consult. Biopsy revealed mild chronic superficial perivascular dermatitis with epidural ulceration and mild superficial perivascular lymphocytic infiltrates in the scant dermis, an example is shown in Figure 2, which is congruent with the patient's negative antineutrophil cytoplasmic antibody (ANCA) results. There was no evidence of leukocytoclastic vasculitis. With these biopsy results, the ID doctor felt that antibiotics were unnecessary, so they were discontinued. During his five-day stay, his leukocytosis, hypokalemia, and AKI resolved, and he was stable for discharge on February 14, 2024, with instructions to follow up with his PCP to get a dermatology referral, as well as wound care instructions.

**Table 1:** Complete blood count - first admission

Complete blood count (CBC)	Reference ranges	Feb 9	Feb 10	Feb 11	Feb 12	Feb 13
White blood cells (WBC)	4,500-11,000/mm^3^	9.6	12.1	12.5	8.7	7.7
Red blood cells (RBC)	20-36 mL/kg	4.31	4.22	3.65	3.57	3.87
Hemoglobin	13.5-17.5 g/dL	12.0	11.6	10.1	9.9	10.9
Hematocrit	41%-53%	36	36	31	30	32
Mean corpuscular volume (MCV)	80-100 μm^3^	84	84	84	84	84
Mean corpuscular hemoglobin (MCH)	25-35 pg/cell	28	28	28	28	28
Red cell distribution width (RDW)	12%-15%	16.1	15.8	16.1	16.0	15.7
Platelet count	150,000-400,000/mm^3^	325	296	254	250	261
Mean platelet volume	8-12 fL	7.7	8.4	7.9	8.0	8.1
Neutrophils	54%-62%	79.1	95.0	94.9	92.2	89.8
Lymphocytes	25%-33%	9.1	3.7	2.8	4.9	6.2
Monocytes	3%-7%	9.3	1.2	2.0	2.8	3.8
Eosinophils	1%-3%	1.6	0	0	0	0
Basophils	0%-0.75%	0.9	0.1	0.3	0.1	0.2
Neutrophils #	1.7-7 x 10^3^/µL	7.6	11.5	11.8	8.1	6.9
Lymphocytes #	0.9-2.9 x 10^3^/µL	0.9	0.4	0.4	0.4	0.5
Monocytes #	0.3-0.9 x 10^3^/µL	0.9	0.1	0.3	0.2	0.3
Eosinophils #	0-0.5 x 10^3^/µL	0.2	0	0	0	0
Basophils #	0-0.2 x 10^3^/µL	0.1	0	0	0	0

**Table 2:** Complete metabolic panel - first admission

Complete metabolic panel (CMP)	Reference ranges	Feb 9	Feb 10	Feb 11	Feb 12	Feb 13
Sodium	136-146 mEq/L	145	144	144	138	138
Potassium	3.5-5 mEq/L	3.0	3.1	3.7	3.8	3.8
Chloride	95-105 mEq/L	98	101	104	103	101
Carbon dioxide	20-31 mmol/L	26.5	26.0	26.5	26.4	27.1
Blood urea nitrogen (BUN)	7-18 mg/dL	29.0	20.0	22.0	24.0	20.0
Creatinine	0.6-1.2 mg/dL	1.7	0.9	0.8	0.8	0.8
Glucose	<140 mg/dL	140	139	147	135	125
Total bilirubin	0.1-1 mg/dL	0.7	N/A	N/A	N/A	N/A
Direct bilirubin	0-0.3 mg/dL	0.2	N/A	N/A	N/A	N/A
Aspartate aminotransferase (AST)	12-38 U/L	21	N/A	N/A	N/A	N/A
Alanine aminotransferase (ALT)	10-40 U/L	21	N/A	N/A	N/A	N/A
Alkaline phosphatase	25-100 U/L	92	N/A	N/A	N/A	N/A
Troponin	<76 ng/L	26	N/A	N/A	N/A	N/A
C-reactive protein (CRP)	0.8-3 mg/L	4.49	N/A	N/A	N/A	N/A
B-type natriuretic peptide (BNP)	0-100 pg/mL	43.58	N/A	N/A	N/A	N/A
Total protein	6-7.8 g/dL	8.4	8.3	N/A	N/A	N/A
Albumin	3.5-5.5 g/dL	3.7	3.4	N/A	N/A	N/A
Globulin	2.3-3.5 g/dL	N/A	4.9	N/A	N/A	N/A
Thyroid-stimulating hormone (TSH)	0.4-4 μU/m	1.17	N/A	N/A	N/A	N/A
Free T4	0.9-1.7 ng/dL	1.09	N/A	N/A	N/A	N/A
Thyroxine (T4)	5-12 μg/dL	8.6	N/A	N/A	N/A	N/A

**Table 3:** Urinalysis - first admission

Urinalysis	Reference ranges	Feb 9
Urine color	Colorless, light yellow, yellow, straw, pale yellow	Light yellow
Urine clarity	Clear	Clear
Urine pH	5-8	6.0
Urine specific gravity	1.005-1.030	1.010
Urine protein	Negative	Negative
Urine ketones	Negative	Negative
Urine blood	Negative	Negative
Urine nitrite	Negative	Negative
Urine bilirubin	Negative, trace	Negative
Urine urobilinogen	<0.2 mg/dL	0.2
Urine leukocyte esterase	Negative	Negative
Urine glucose	Negative	Negative

**Table 4:** Coagulation studies - first admission

Coagulation studies	Reference ranges	Feb 9
Prothrombin time (PT)	11-15 seconds	10.3
International normalized ratio (INR)	0.8-1.1	1.0
Partial thromboplastin time (PTT)	25-40 seconds	26.6
Fibrinogen	200-400 mg/dL	460
D-dimer	0.5 mg/L	3,480
ADAMTS13 antibody	<12 U/mL	8

**Table 5:** Serology results - first admission

Virology	Reference ranges	Feb 9
Hepatitis A IgM antibody	Non-reactive	Non-reactive
Hepatitis B surface antigen	Non-reactive	Non-reactive
Hepatitis B core IgM antibody	Non-reactive	Non-reactive
Hepatitis C antibody	Non-reactive	Non-reactive
Rapid HIV 1/2 antibody	Negative	Negative
Influenza type A antibody	Negative	Negative
Influenza type B antibody	Negative	Negative
Rapid SARS-CoV-2 antigen	Negative	Negative

**Table 6:** Toxicology results - first admission

Toxicology	Reference ranges	Feb 9
Urine opiates	Negative	Negative
Urine fentanyl	Negative	Negative
Urine barbiturates	Negative	Negative
Urine phencyclidine	Negative	Negative
Urine amphetamines	Negative	Positive
Urine benzodiazepines	Negative	Negative
Urine cocaine	Negative	Negative
Urine cannabinoids	Negative	Negative

**Figure 3:**
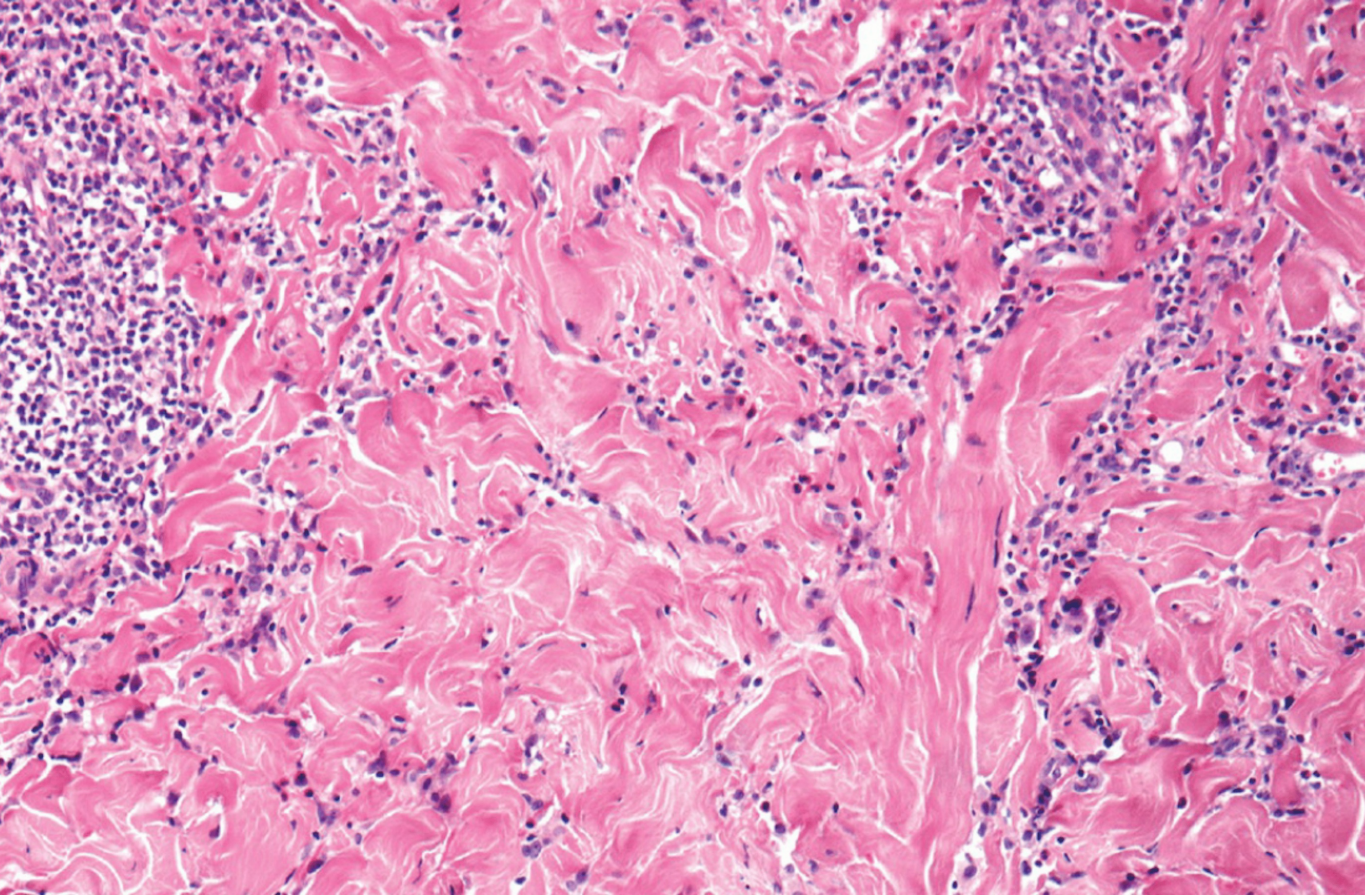
Example 2 of biopsy of superficial perivascular dermatitis at 10x magnification

On February 23, 2024, the patient was readmitted to the hospital with bilateral lower extremity edema with burning and shooting sensations in the legs. Physical exam at this time was similar to his previous admission, however the purpura in his upper extremities had resolved. He was started on Meropenem and Vancomycin empirically for the skin rash. A repeat skin biopsy showed mild chronic superficial perivascular dermatitis with epidermal ulceration, another example can be seen in Figure 3. Repeat wound and blood cultures were taken and both MRSA and *Enterobacter cloacae* were in the wound. Labs showed that he did develop leukocytosis, but again this is likely due to steroids that he had been given. His d-dimer remained elevated but improved from the previous admission. He continued to have normocytic anemia but otherwise, his labs were unremarkable (Tables 7-11). He was discharged on February 16 with Levofloxacin based on sensitivity results. He was informed to follow up at the continuity clinic and his dermatology referral was pending authorization at this time.

**Table 7:** Complete blood count - second admission

Complete blood count (CBC)	Reference ranges	Feb 23	Feb 24	Feb 25	Feb 26
White blood cells (WBC)	4,500-11,000/mm^3^	10.0	8.2	14.9	8.9
Red blood cells (RBC)	20-36 mL/kg	4.05	4.04	4.05	4.00
Hemoglobin	13.5-17.5 g/dL	11.3	11.2	11.2	11.3
Hematocrit	41%-53%	34	34	34	34
Mean corpuscular volume (MCV)	80-100 μm^3^	85	85	84	84
Mean corpuscular hemoglobin (MCH)	25-35 pg/cell	28	28	28	28
Red cell distribution width (RDW)	12%-15%	16.9	17.3	16.7	17.3
Platelet count	150,000-400,000/mm^3^	305	263	296	254
Mean platelet volume (MPV)	8-12 fL	8.0	7.1	7.3	7.4
Neutrophils	54%-62%	N/A	96.0	91.1	79.4
Lymphocytes	25%-33%	N/A	2.8	3.3	14.0
Monocytes	3%-7%	N/A	0.7	5.2	6.1
Eosinophils	1%-3%	N/A	0.2	0	0.2
Basophils	0%-0.75%	N/A	0.3	0.4	0.3
Neutrophils #	1.7-7 x 10^3^/µL	N/A	7.8	13.6	7.0
Lymphocytes #	0.9-2.9 x 10^3^/µL	N/A	0.2	0.5	1.2
Monocytes #	0.3-0.9 x 10^3^/µL	N/A	0.1	0.8	0.5
Eosinophils #	0-0.5 x 10^3^/µL	N/A	0	0	0
Basophils #	0-0.2 x10^3^/µL	N/A	0	0.1	0

**Table 8:** Complete metabolic panel - second admission

Complete metabolic panel (CMP)	Reference ranges	Feb 23	Feb 24	Feb 25	Feb 26
Sodium	136-146 mEq/L	140	N/A	142	142
Potassium	3.5-5 mEq/L	3.7	N/A	3.3	3.6
Chloride	95-105 mEq/L	101	N/A	100	106
Carbon dioxide	20-31 mmol/L	27.1	N/A	26.7	29.7
Blood urea nitrogen (BUN)	7-18 mg/dL	27.0	N/A	25.0	24.0
Creatinine	0.6-1.2 mg/dL	0.8	N/A	0.9	0.8
Glucose	<140 mg/dL	103	N/A	160	95
Hemoglobin A1c	4%-5.6%	N/A	5.3	N/A	N/A

**Table 9:** Urinalysis - second admission

Urinalysis	Reference ranges	Feb 24
Urine color	Colorless, light yellow, yellow, straw, pale yellow	Light yellow
Urine clarity	Clear	Clear
Urine pH	5-8	6.0
Urine specific gravity	1.005-1.030	1.015
Urine protein	Negative	Negative
Urine ketones	Negative	Negative
Urine blood	Negative	Negative
Urine nitrite	Negative	Negative
Urine bilirubin	Negative, trace	Negative
Urine urobilinogen	<0.2 mg/dL	0.2
Urine leukocyte esterase	Negative	Negative
Urine glucose	Negative	Negative

**Table 10:** Coagulation studies - second admission

Coagulation studies	Reference ranges	Feb 24
Prothrombin time (PT)	11-15 seconds	12.6
International normalized ratio (INR)	0.8-1.1	1.2
Partial thromboplastin time (PTT)	25-40 seconds	30.5
Fibrinogen	200-400 mg/dL	443
D-dimer	0.5 mg/L	1,420

**Table 11:** Toxicology results - second admission

Toxicology	Reference ranges	Feb 24
Urine opiates	Negative	Negative
Urine fentanyl	Negative	Negative
Urine barbiturates	Negative	Negative
Urine phencyclidine	Negative	Negative
Urine amphetamines	Negative	Positive
Urine benzodiazepines	Negative	Negative
Urine cocaine	Negative	Negative
Urine cannabinoids	Negative	Negative

On May 24, he was readmitted for similar lower extremity swelling and painful wounds/rash. He reported finishing his course of antibiotics but admitted to poor outpatient follow-up. During this admission, Bactrim was started based on previous cultures. Podiatry was consulted to discuss wound care and any further treatment that the patient may need. X-rays of the right ankle were ordered to rule out osteomyelitis and they showed soft tissue irregularity of the distal right lower leg with soft tissue swelling. Podiatry had no recommendations at this time but did want to follow up with him outpatient. Labs during this admission were unremarkable (Tables 12-16). Repeat wound cultures grew MRSA, *Escherichia coli*, and *Klebsiella oxytoca* which were all sensitive to Bactrim. He was discharged on March 26 with Bactrim and instructions to follow up with the continuity clinic and podiatry.

**Table 12:** Complete blood count - third admission

Complete blood count (CBC)	Reference ranges	May 24	May 25	May 26
White blood cells (WBC)	4,500-11,000/mm^3^	7.0	7.9	6.9
Red blood cells (RBC)	20-36 mL/kg	4.85	5.55	5.17
Hemoglobin	13.5-17.5 g/dL	13.7	15.6	14.3
Hematocrit	41%-53%	40	47	44
Mean corpuscular volume (MCV)	80-100 μm^3^	83	85	85
Mean corpuscular hemoglobin (MCH)	25-35 pg/cell	28	28	28
Red cell distribution width (RDW)	12%-15%	15.7	16.3	16.5
Platelet count	150,000-400,000/mm^3^	280	320	298
Mean platelet volume (MPV)	8-12 fL	7.4	8.3	7.9

**Table 13:** Complete metabolic panel - third admission

Complete metabolic count (CMP)	Reference ranges	May 25	May 26
Sodium	136-146 mEq/L	144	144
Potassium	3.5-5 mEq/L	3.8	3.9
Chloride	95-105 mEq/L	105	105
Carbon dioxide	20-31 mmol/L	27.7	25.2
Blood urea nitrogen (BUN)	7-18 mg/dL	14.0	21.0
Creatinine	0.6-1.2 mg/dL	0.8	0.9
Glucose	<140 mg/dL	99	91
Calcium	8.4-10.2 mg/dL	9.3	9.1
Phosphorus	3-4.5 mg/dL	3.5	4.0
Magnesium	1.5-2 mg/dL	2.1	2.0
Total bilirubin	0.1-1 mg/dL	1.00	1.0
Aspartate aminotransferase (AST)	12-38 U/L	16	19
Alanine aminotransferase (ALT)	10-40 U/L	24	22
Alkaline phosphatase	25-100 U/L	109	98

**Table 14:** Urinalysis - third admission

Urinalysis	Reference ranges	May 24
Urine color	Colorless, light yellow, yellow, straw, pale yellow	Light yellow
Urine clarity	Clear	Clear
Urine pH	5-8	7.5
Urine specific gravity	1.005-1.030	1.010
Urine protein	Negative	Negative
Urine ketones	Negative	Negative
Urine blood	Negative	Negative
Urine nitrite	Negative	Negative
Urine bilirubin	Negative, trace	Negative
Urine urobilinogen	<0.2 mg/dL	0.2
Urine leukocyte esterase	Negative	Negative
Urine glucose	Negative	Negative

**Table 15:** Coagulation studies - third admission

Coagulation studies	Reference ranges	May 24
Prothrombin time (PT)	11-15 seconds	10.4
International normalized ratio (INR)	0.8-1.1	1.0
Partial thromboplastin time (PTT)	25-40 seconds	26.6
D-dimer	0.5 mg/L	358

**Table 16:** Toxicology results - third admission

Toxicology	Reference ranges	Feb 24
Urine opiates	Negative	Negative
Urine fentanyl	Negative	Negative
Urine barbiturates	Negative	Negative
Urine phencyclidine	Negative	Negative
Urine amphetamines	Negative	Positive
Urine benzodiazepines	Negative	Negative
Urine cocaine	Negative	Negative
Urine cannabinoids	Negative	Negative

## Discussion

Superficial perivascular dermatitis is an inflammation of the skin characterized by inflammatory cells surrounding, invading, and damaging blood vessels [4]. This term is used as an umbrella term for a variety of different skin conditions, as it is a diagnosis of exclusion. Extensive workup is needed to exclude other differentials, including a thorough history taking of the patient, lab work, and biopsy.

The most common type of superficial perivascular dermatitis has a predominant lymphocytic infiltrate [1], which was seen in our patient’s first biopsy report. However, delayed hypersensitivity reactions present within a few days and usually self-resolves when the trigger is removed [5]. Alternatively, urticarial dermatitis presents with erythema, pruritis, and possibly plaques and papules [6]. Other causes of superficial perivascular dermatitis are drug interactions, urticaria, and viral exanthema [4]. Drug-induced hypersensitivity reactions can cause a lymphocytic dominant superficial perivascular dermatitis, but biopsy results may show some eosinophils scattered throughout as well, although this is not always the case [4]. This can look similar to lymphocytic dominant superficial vascular dermatitis; however, the biopsy also shows spongiosis, eosinophils, and sometimes parakeratosis indicating plaque formation [4]. Viral exanthema can be caused by a variety of viruses and is usually lymphocytic in nature, but viral serology testing would be positive for whatever the patient was exposed to [4]. Biopsies for superficial perivascular dermatitis and delayed hypersensitivity reactions can appear very similar, so it is important to get a thorough history to distinguish between the two [7]. Superficial perivascular dermatitis presents with erythema, pruritis, pain, and sometimes edema and swelling. When exposed to the trigger for a long period of time, there can be plaque formation and scaling of the skin [8] because of this similarity, it is important to get a biopsy.

In this patient, there were no clear identifying triggers, so it is likely that his dermatitis was idiopathic in nature, although the patient was positive for urine methamphetamines, other differentials should not be excluded such as linear IgA, dermatitis herpetiformis, pemphigoid, ANCA-mediated vasculitis. He denied any recent illnesses, was not prescribed any new medications, was not sexually active, and was not immunologically compromised. He only admitted to medication noncompliance and amphetamine use, both of which exacerbated his disease and prevented the healing process. Due to the five-month duration of the rash, it is unlikely that this was a case of viral exanthem, as viral exanthem is usually acute and resolves on its own [9]. He did not report any known allergies, and no eosinophils were seen on the biopsy, so it is also unlikely that this was a case of urticaria.

Management of perivascular dermatitis depends on the cause and subtype, but generally symptomatic care and treating the underlying cause will resolve the rash. Keeping the rash clean and moisturized, avoiding any exacerbating factors, and managing stress can be helpful [8]. Antibiotics can be used if there is a concurrent infection; however, they are not needed if there is no concern for infection. Topical corticosteroids can be used for inflammation [8].

This case shows that not only is perivascular dermatitis difficult to manage due to the multiple variables that could be causing the disease but also stresses the importance of close follow-up and compliance with medical instructions at discharge. Without compliance and close follow-up, his dermatitis is likely to persist. Treating his underlying infection is an important step in treatment, as it is likely that the infection is preventing wound healing. Noncompliance to antibiotics may result in an infection that becomes resistant to antibiotics. Without the proper wound care at home, the rash is likely to persist and become reinfected. Following up with his primary care provider and other specialists is important to monitor the rash and provide any necessary changes to treatment.

## Conclusions

Superficial perivascular dermatitis is the most common inflammatory skin disease; however, it can be difficult to narrow down differentials due to similar presentation across multiple diagnoses. Proper diagnostic studies are crucial for diagnosis, including getting a biopsy sample deep enough to examine. Identifying the underlying cause of superficial perivascular dermatitis can pose a challenge, as in this case. The common triggers of perivascular dermatitis, including insect bites, viral infection, and any allergic exposures, are unlikely in this patient. Although unlikely there are several viruses associated with purpuric rashes and non-hemorrhagic blisters that can be considered, these include but are not limited to measles, rubella, parvo, varicella, and many others. Additionally, although the patient did not test positive for cocaine on the urine drug screen, cocaine-levamisole-induced vasculopathy cannot fully be excluded as testing positive for cocaine is not always an indicator. This patient likely had a case of idiopathic superficial perivascular dermatitis complicated by infection and noncompliance; however, it is important to understand that there can be other differentials despite this patient's particular symptoms and presentation. Early recognition and management of superficial perivascular dermatitis is important because it can help with the patient’s pain and discomfort, as well as increase the likelihood of resolution. Compliance with treatment and follow-up is also important to decrease the likelihood of persistence and recurrence.
